# Dilated Cardiomyopathy Due to Alimentary Iron Deficiency

**DOI:** 10.3390/children11020196

**Published:** 2024-02-04

**Authors:** Leonie Dewein, Andrea Kresz, Jochen Essers, Peter Bride, Michael Kaestner, Christian Apitz

**Affiliations:** 1 Division of Pediatric Cardiology, University Children Hospital, Eythstr. 24, 89075 Ulm, Germany; 2 Division of Neonatology and Intensive Medicine, University Children Hospital, Eythstr. 24, 89075 Ulm, Germany

**Keywords:** nutrition, cardiomyopathy, paediatric cardiology, anaemia, iron

## Abstract

Dilated cardiomyopathy (DCM) is a severe condition, characterised by left ventricular dilation and systolic dysfunction, necessitating heart transplantation when all other treatment options fail. This case report describes a 2-year-old girl initially presenting with oedema, listlessness, and severe iron deficiency anaemia. She was diagnosed with DCM. Extensive diagnostic workup ruled out other causes, leading to the suspicion of DCM due to alimentary iron deficiency. This was confirmed by the parents’ report that the girl was fed almost exclusively with low-fat cow’s milk. Prompt treatment, including packed red cell transfusion, iron supplementation, and heart failure medications (diuretics, ACE inhibitors, beta blockers, and aldosterone antagonists), resulted in significant improvement in cardiac function within days. This report demonstrates the potential risks of alimentary iron deficiency, the most common cause of microcytic hypochromic anaemia in young children, which might even result in the development of life-threatening cardiac dysfunction in extreme cases.

## 1. Introduction

Dilated cardiomyopathy (DCM) is characterised by the dilation of the left ventricle accompanied by systolic dysfunction. DCM is a serious disease with high morbidity and mortality [1,2,3]. In approximately 40% of cases with DCM, the only treatment option is a heart transplantation [1].

DCM in children is a complex condition with multifactorial causes. The causes of DCM in children are diverse and often interconnected. A holistic approach to diagnosis, considering infectious, environmental, metabolic, and genetic factors, is essential for effective management and improving outcomes in paediatric patients with DCM.

One significant contributor of DCM is genetic predisposition, where inherited mutations in specific genes can weaken the heart muscle over time. These genetic factors often play a role in familial cases of DCM, emphasizing the importance of family history in diagnosis. Viral infections also pose a risk, as certain viruses can directly attack and damage the heart muscle, leading to dilation. Coxsackievirus and adenovirus are among the viruses associated with paediatric DCM, highlighting the need for vigilant monitoring after viral illnesses. The exposure to toxins, such as certain medications, chemicals, or environmental factors, can contribute to DCM. Understanding potential environmental triggers is essential for preventing and managing cases that are related to toxic exposure. Metabolic disorders, including those affecting the thyroid or the storage of essential nutrients, may impact cardiac function in children. Disorders like glycogen storage diseases or mitochondrial disorders can manifest as DCM, emphasizing the necessity of metabolic assessments in diagnosis. In more than half of cases, the reason for the development of DCM remains unclear [1]. This underscores the challenges in unravelling the intricate web of factors leading to paediatric DCM. Timely diagnosis through imaging, laboratory testing, and thorough medical evaluation is pivotal for implementing appropriate treatment plans that are tailored to the specific underlying causes, when possible.

## 2. Detailed Case Description

We report on a 2-year-old girl (weight 12.2 kg (Z-score +0.24), height 86.5 cm (Z-score +0.18)), who initially presented to an external hospital with peripheral oedema, as well as increasing listlessness and shortness of breath. There, laboratory tests revealed hypochromic, microcytic anaemia with a haemoglobin concentration (Hb) of 1.4 mg/dL (norm: 12.3–15.3 g/dL). Further lab values included the following: Erythrocytes 1.16 Mio/L (norm: 4.3–6.3 Mio/L), Hct 7.1% (norm: 32–40%), MCV 62 fl (norm: 74–102 fl), MCH 12.9 pg/cell (norm: 23–31 pg/cell), and MCHC 20.9 g/dL (norm: 26–34 g/dL). Immediately, transfusion of red blood cells was initiated, and subsequently, the child was transferred to our hospital.

**Figure 1:**
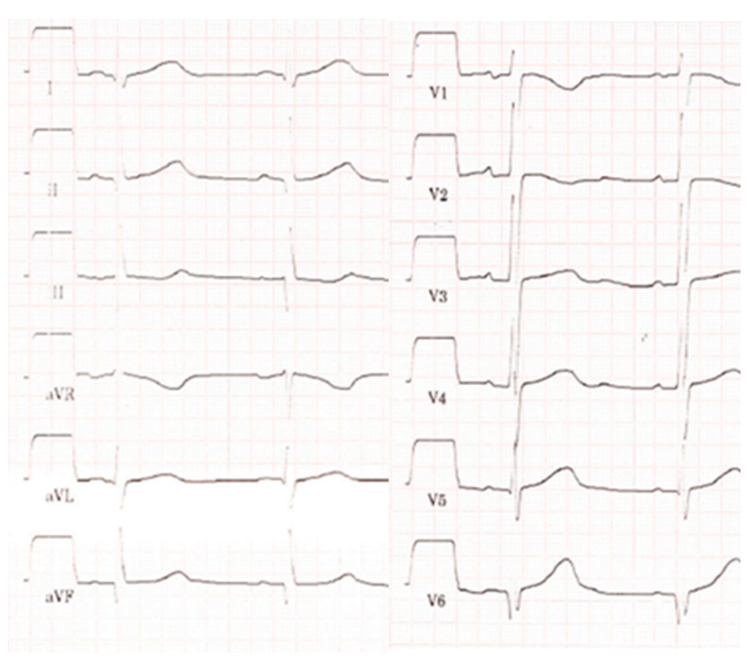
Electrocardiogram showing sinus rhythm, time intervals in the normal range, prominent Q-waves in III, aVF, and V6.

On clinical examination, the girl presented with very pale skin colour, peripheral oedema, marked hepatomegaly (8 cm below the costal arch), and tachycardia with a heart rate of 142/min. After an additional red blood cell transfusion, the haemoglobin level increased adequately to 7.4 mg/dL. Electrocardiogram showed sinus rhythm with normal time intervals and without signs of relevant ischemia or hypertrophy (Figure 1).

Further blood work revealed low iron content of 7.4 µmol/L (norm: 5.8–34.5 µmol/L), and transferrin saturation of 9% (norm: 7–44%), reduced ferritin of <5 µg/L (norm: 6–67 µg/L), and increased transferrin of 3.2 g/L (norm: 1.9–3.0 g/L), and soluble transferrin receptor of 17.59 mg/L (norm: 1.9–4.4 mg/L). Ferritin Index was 25.2 (norm: 0.0–3.2).

Additional laboratory tests showed no evidence of bacterial or viral infection (including CMV, EBV, HHV6, and parvovirus B19). There was no evidence of vitamin B12 or folic acid deficiency. For evaluation of the observed hepatomegaly, abdominal ultrasound was performed and did not reveal any structural organic abnormalities. There was no evidence of active bleeding as the cause of the anaemia.

**Figure 2:**
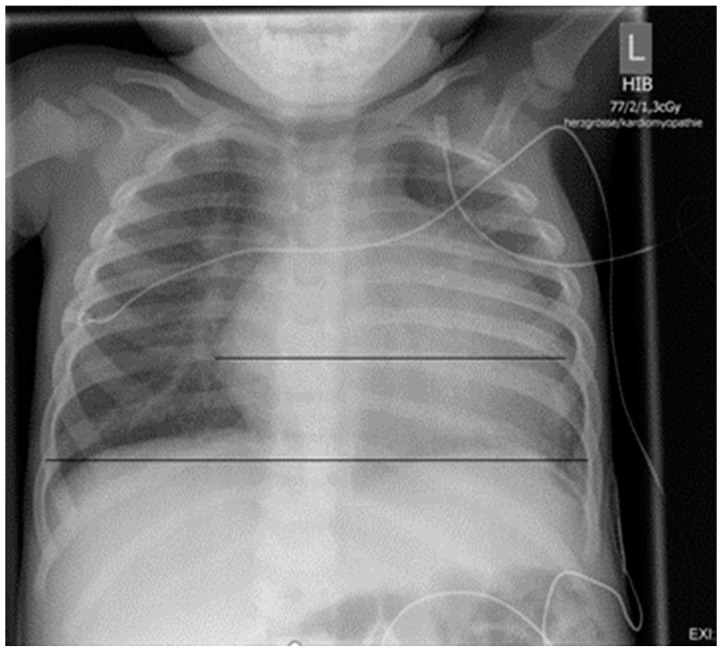
Chest X-ray on admission: cardiac silhouette is clearly enlarged (HTQ 0.64).

In order to investigate for reasons of the observed shortness of breath, an X-ray examination of the thorax was performed, which revealed a clearly enlarged cardiac silhouette (Figure 2). Additional laboratory test of cardiac biomarkers revealed elevated NT-proBNP of 14258 pg/mL (norm: <320 pg/mL), while Troponin was normal.

**Figure 3:**
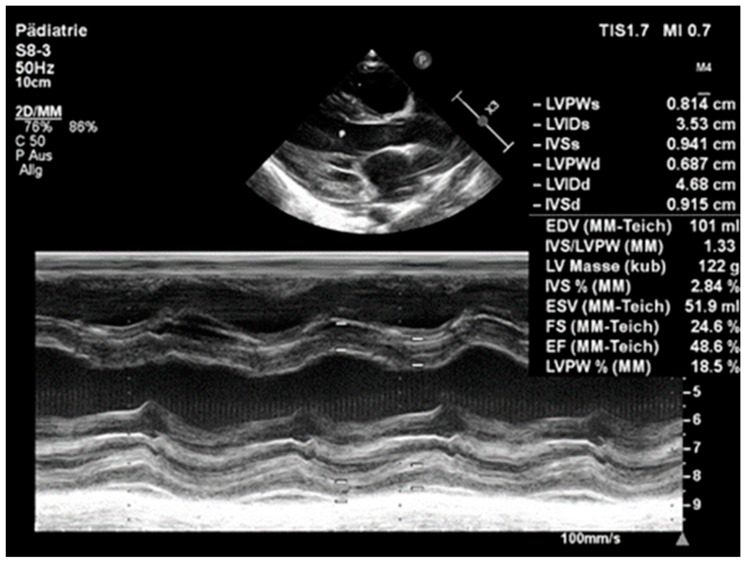
Echocardiography on admission: left ventricle is significantly enlarged (LV enddiastolic diameter of 4.68 (Z-score +4.7) and reduced systolic function (EF 48.6%).

On echocardiography, left ventricular function was reduced, with a shortening fraction of 24.6% (ejection fraction: 48.6%), and the left ventricle was markedly dilated, with an end-diastolic diameter of 46.8 mm (Z-score +4.7), and hypertrophied with an end-diastolic wall thickness of septal wall of 9.1 mm (Z-score +3.8) and left ventricular posterior wall of 6.9 mm (Z-score +2.9) (Figure 3). There was mild mitral valve regurgitation and aortic valve regurgitation, most likely as a result of the pronounced dilatation of the left ventricle. Anomalous origin of the left coronary artery as well as aortic coarctation could be excluded by echocardiography.

Anticongestive treatment with diuretics (furosemide), ACE inhibitor (lisinopril), beta blocker (bisoprolol), and aldosterone antagonist (spironolactone) was started in addition to two transfusions. Cardiac function improved significantly within a few days. After two weeks, shortening fraction was normalised to 31% (EF 60%), and LV enddiastolic diameter improved to 38 mm (Z-score +2.8). Haemoglobin values reached low normal level after the two transfusions and remained stable with oral iron supplementation of 3 × 30 mg. With replenished iron stores being confirmed by laboratory tests, oral iron supplementation could be stopped after 10 days.

There was no family history of cardiomyopathy. After careful taking of repeated medical history, the mother admitted that the girl was fed almost exclusively with low-fat cow’s milk. Since according to the current WHO guidelines, iron-rich complementary foods should be introduced between the 4th and 6th month of life, and a child aged 2 years should be fed a balanced mixed diet/family diet and should only receive cow’s milk as add-on, the observed severe iron deficiency was obviously caused by malnutrition [4,5]. Consequently, the family received nutritional counselling and detailed information regarding appropriate food combinations.

## 3. Discussion

To the best of our knowledge, this is the first report of a DCM that was diagnosed in an infant due to severe iron deficiency anaemia, induced by malnutrition through almost exclusively feeding with low-fat cow’s milk. It was previously demonstrated that the early introduction of cow’s milk has a negative effect on the iron status of infants [2,6,7]. On the other hand, iron fortification of milk or other foods in infants or the elderly helps significantly reduce the serious negative consequences of iron deficiency anaemia [2,6,7,8].

If left untreated, iron deficiency not only impairs psychomotor development, but may also lead to life-threatening heart disease, such as dilated cardiomyopathy with impaired left ventricular function and heart failure [2,9]. An Hb < 7 g/dL initially leads to hyperdynamic contractility of the left ventricle and, with an Hb < 5 g/dL, most likely to dilated cardiomyopathy [2,3]. The pathogenesis has not yet been conclusively clarified, but in a mouse model, the study by Chung et al. showed that iron deficiency anaemia leads to a contraction deficit of the heart. Increased markers for hypoxia and iron deficiency were detected in the hearts of the mice with iron deficiency, whereby the tissue hypoxia appears to be the result of the anaemia. Oxygen and iron deficiency resulted in down-regulation of RYR channels, leading to myocyte dysfunction via the Ca^2+^ signalling pathway and, thus, to cardiomyopathy. The effects on Ca^2+^ signalling occur via two molecules. These have previously been linked to the development of heart failure, which may explain why patients with an iron deficiency are more susceptible to increasing contractile dysfunction [2,10,11]. Two doses of intravenous iron alone reversed the changes. In this study, however, the mice were only fed a diet that caused moderate anaemia. Accordingly, the extent of the contractile dysfunction was moderate, and the reduction in the ejection fraction was not in the range of decompensated heart failure. It can therefore be assumed that only very severe anaemia is likely to lead to heart failure [10].

Overall, the outcome of cardiomyopathies that are caused by malnutrition is obviously better with adequate timely treatment than with other cardiomyopathies [1,10,12]. If the iron deficiency is sufficiently replaced, the cardiomyopathy is expected to be completely reversible. If it does not reverse, other causes should be reconsidered [13]. As in the presented case, an improvement in cardiac function and a reduction in the end-diastolic left ventricular diameter can frequently be observed within a few days, but complete normalisation may need several months. Until normalisation, the heart should be supported with anticongestive therapy [1,10,12].

### 3.1. Alimentary Iron Deficiency

Alimentary iron deficiency is well known to be the most common cause of microcytic, hypochromic anaemia between 6 months and 3 years of age [14,15,16]. It affects more than 40% of children worldwide and a quarter of children under the age of 5 years in Europe [17]. Overall, premature babies, infants, young children, female teenagers, and women in child-bearing age, as well as pregnant women, are at risk of iron deficiency anaemia [15]. Previous studies have also shown that a low level of maternal education is associated with a higher prevalence of iron deficiency anaemia [2,6]. Severe iron deficiency anaemia during pregnancy increases the risk of premature birth, a low birth weight, as well as morbidity and mortality of the newborn [15,17]. Iron is essential for neuronal metabolism. The development of cognitive and motor skills, as well as social behaviour, is limited in iron deficiency [2,9,18]. The damage caused by early iron deficiency in childhood is not always reversible [2,9,10,12,18]. The need for iron is highest in the second year of life due to growth [6,15,16]. This should be covered with iron-rich foods such as meat or pulses.

### 3.2. Dietary Recommendations for Newborns and Infants

The current WHO guidelines recommend breastfeeding or, if necessary, giving breast milk by bottle from birth until at least 6 months of age. Exclusive breastfeeding up to 6 months of age has many advantages for the infant, but also for the mother. Children are well protected against infectious diseases, especially gastrointestinal infections. An early initiation of breastfeeding within an hour of birth reduces neonatal mortality. From the age of 6–7 months, the energy and nutrient balance exceeds the supply of breast milk. The introduction of complementary foods at this time is therefore important to enable timely growth. Babies at this age are also developmentally ready for porridge or soft foods. The current guidelines recommend initially introducing a vegetable, potato, and meat porridge for lunch. Four weeks later, another fruit and cereal porridge should be introduced as an afternoon snack, followed four weeks later by a milk and cereal porridge. During this time, the child should continue to be breastfed. As soon as three porridge meals have been introduced, water should also be given at mealtimes. It is important to introduce more and more different foods to develop flavour. Furthermore, more and more chunky foods should be introduced to promote the learning of the swallowing act. Bread meals should be added from the age of 9 months. From the age of 1 year, a balanced mixed diet/family diet is recommended, as is also usual for adults [4,5].

Infants aged 6–8 months should receive 2–3 meals per day, infants aged 9–23 months 3–4 meals per day. One or two additional snacks are generally necessary for children. The WHO currently recommends continuing breastfeeding on demand up to the age of 2 years or even beyond. However, it is important not to breastfeed exclusively, as this can lead to a pronounced iron deficiency, as in our case. Breast milk is an important source of energy and nutrients, especially during illness, and in malnourished children, the mortality rate is significantly reduced. Prolonged breastfeeding also shows clear benefits for the mother, as the risk of ovarian and breast cancer is reduced due to the altered hormone balance [4,5].

### 3.3. Risks of Special Diets

Vegetarian and vegan diets are very popular currently, but special attention must be paid to growing children. While a well-planned vegetarian diet for infants and toddlers is not a major problem, a vegan diet must be well planned, supplemented, and controlled in order to prevent the risk of potentially serious nutrient deficiencies in childhood. The implementation of such a diet requires a sound knowledge of nutritional science on the part of the carer [19].

A balanced ovo-lacto-vegetarian diet can provide almost all the nutrients that a growing organism needs. Only the intake of iron, long-chain omega-3 fatty omega-3 fatty acids, and vitamin B12 may be too low [20]. These deficiencies can be prevented by targeted dietary measures. In addition, vitamin D must be supplemented in the first three years of life [19].

Vegan nutrition is characterised by a rich coverage of β-carotene; folate; niacin; vitamins B1, B6, and C; potassium; and magnesium, as well as dietary fibre and secondary plant substances. The latter are primarily recognised as protective modulators in the pathogenesis of inflammatory and carcinogenic processes. On the other hand, a diet that completely avoids foods of animal origin can be potentially critical in terms of energy, protein quality, long-chain omega-3 fatty acids, iron, zinc, iodine, calcium, vitamin D, riboflavin, and especially, vitamin B1. Knowledge of these potentially critical nutrients allows parents who are planning a vegan diet for themselves and their children to make a conscious choice of foods and nutritional supplements [19].

## 4. Conclusions

Iron deficiency in children is still a problem that should not be underestimated and may have long-term consequences. In order to avoid deficiencies and possibly fatal outcomes, iron-rich complementary foods should be introduced between the 4th and 6th month of life, and the iron status should be checked by laboratory chemistry if necessary. In particular, families on a vegetarian diet should be advised of appropriate food combinations, ideally by a professional nutritionist, in order to increase the poorer bioavailability of non-haem iron. Should a pronounced iron deficiency anaemia nevertheless occur, echocardiography is essential to rule out or detect cardiomyopathy at an early stage. In the presence of a malnutritive anaemia-related cardiomyopathy, early and rapid iron supplementation should be given, including the administration of packed red cells if necessary, and heart failure therapy should be started. With adequate treatment, a rapid normalisation of cardiac function can usually be achieved.

## Data Availability

Data are contained within the article.
